# Exposure to 1.8 GHz electromagnetic fields affects morphology, DNA-related Raman spectra and mitochondrial functions in human lympho-monocytes

**DOI:** 10.1371/journal.pone.0192894

**Published:** 2018-02-20

**Authors:** M. Lasalvia, R. Scrima, G. Perna, C. Piccoli, N. Capitanio, P. F. Biagi, L. Schiavulli, T. Ligonzo, M. Centra, G. Casamassima, A. Ermini, V. Capozzi

**Affiliations:** 1 Dipartimento di Medicina Clinica e Sperimentale, Università di Foggia, Foggia, Italy; 2 Istituto Nazionale di Fisica Nucleare–sezione di Bari, Bari, Italy; 3 Dipartimento Interateneo di Fisica, Università di Bari, Bari, Italy; 4 Banca del sangue, Ospedali Riuniti di Foggia, Foggia, Italy; 5 Dipartimento di Ingegneria Industriale, Università di Tor Vergata, Roma, Italy; Columbia University, UNITED STATES

## Abstract

Blood is a fluid connective tissue of human body, where it plays vital functions for the nutrition, defense and well-being of the organism. When circulating in peripheral districts, it is exposed to some physical stresses coming from outside the human body, as electromagnetic fields (EMFs) which can cross the skin. Such fields may interact with biomolecules possibly inducing non thermal-mediated biological effects at the cellular level. In this study, the occurrence of biochemical/biological modifications in human peripheral blood lympho-monocytes exposed in a reverberation chamber for times ranging from 1 to 20 h to EMFs at 1.8 GHz frequency and 200 V/m electric field strength was investigated. Morphological analysis of adherent cells unveiled, in some of these, appearance of an enlarged and deformed shape after EMFs exposure. Raman spectra of the nuclear compartment of cells exposed to EMFs revealed the onset of biochemical modifications, mainly consisting in the reduction of the DNA backbone-linked vibrational modes. Respirometric measurements of mitochondrial activity in intact lympho-monocytes resulted in increase of the resting oxygen consumption rate after 20 h of exposure, which was coupled to a significant increase of the FoF1-ATP synthase-related oxygen consumption. Notably, at lower time-intervals of EMFs exposure (i.e. 5 and 12 h) a large increase of the proton leak-related respiration was observed which, however, recovered at control levels after 20 h exposure. Confocal microscopy analysis of the mitochondrial membrane potential supported the respiratory activities whereas no significant variations in the mitochondrial mass/morphology was observed in EMFs-exposed lympho-monocytes. Finally, altered redox homeostasis was shown in EMFs-exposed lympho-monocytes, which progressed differently in nucleated cellular subsets. This results suggest the occurrence of adaptive mechanisms put in action, likely *via* redox signaling, to compensate for early impairments of the oxidative phosphorylation system caused by exposure to EMFs. Overall the data presented warn for health safety of people involved in long-term exposure to electromagnetic fields, although further studies are required to pinpoint the leukocyte cellular subset(s) selectively targeted by the EMFs action and the mechanisms by which it is achieved.

## Introduction

The exposure of humans to electromagnetic fields (EMFs) enormously increased during the last century and it is still increasing today as a consequence of the industrial and technical development, which involves generation of artificial EMFs sources (radio and television stations and receivers, radar, computers, Wi-Fi antennas, mobile phones, microwave ovens, and many devices used in medicine and industry). In particular, the exposure to EMFs (from 300 MHz to 300 GHz) is extensively increasing due to the huge use of wireless communications devices working in such a frequency range. The first effect of such exposure consists in the local heating of tissues located inside the penetration depth of radiation (thermal effect), as a consequence of energy transfer from EMFs to the oscillating charges inside the biological tissues. However, the increase of temperature is counteracted by physiological mechanisms of heat dispersion and, therefore, it can be considered as a negligible effect with no dangerous risks involved. The non-thermal-related effects of EMFs on biomolecular structures have been largely investigated in the latest years. Nevertheless, because of controversial and discrepant results, this issue is still object of scientific debate. This subject is still largely investigated, especially by *in vitro* experiments, where some experimental parameters can be changed and easily controlled.

Xu et al. found that EMFs induces DNA damage in human skin fibroblasts, but such a damage did not result in significant cellular dysfunctions [1]. Other investigations have concluded that the data from genotoxicity studies do not support a causal relationship between EMFs exposure of mobile phones and the incidence of brain cancer or other tumors of the head [2]. In particular, de Pomerai et al. found that EMFs radiation can alter protein conformation without heating effects [3]. Esmekaya et al. [4] reported experiments about mutagenic and morphologic effects of 1.8 GHz radiofrequency in human cultured peripheral blood lymphocytes at a Specific Absorption Rate (SAR) average of 0.21 W/Kg. Effects such as destruction of organelle and nucleus structures, cytoplasm lysis and destruction of membrane integrity resulted more pronounced with increasing exposure time up to 48 h.

Among biological tissues, blood is of interest for investigation of EMFs effects because it is present in every human body district, both in those located deeper and in the shallower ones, just below the skin. So, blood is mainly involved in the environmental EMFs exposure. Among the different blood components, monocytes play a key role, as part of the innate immune system, to recognize and eliminate pathogens. So, they are fundamental components in the protection of the human organism against infections. Monocytes are mononuclear cells with a diameter ranging from 10 to 15 μm and account for about 10% of total blood leukocytes. They can migrate from the bloodstream into different tissues of the human body and differentiate in macrophages, dendritic cells and microglial cells depending on the tissue in which they migrate. Macrophages have the ability of phagocytosis and they produce cytokines capable of activating other inflammatory cells. Moreover, macrophages secrete enzymes and growth factors, such as the platelet growth factor, which stimulates the growth and activity of fibroblasts and endothelial cells, thus contributing to tissue repairing. Lymphocytes are a major subtype of leukocytes in a vertebrate's immune system. Lymphocytes include natural killer cells (NK cells which function in cell-mediated, cytotoxic innate immunity), T cells (for cell-mediated, cytotoxic adaptive immunity), and B cells (for humoral, antibody-driven adaptive immunity).

In a previous study [5], we reported that EMFs at 1.8 GHz are able to produce morphological alterations in human peripheral blood leukocytes, mainly consisting in the increase of the cell surface area. Several authors report that human peripheral blood lymphocytes which have been pre-exposed to non-ionizing EMFs exhibit an adaptive response by resisting the induction of genetic damage from subsequent exposure to ionizing radiation [6–8].

As many blood diseases are linked to biochemical changes in the cells composing the blood, analytical methods are suitable to investigate the biochemical effects due to blood exposure to EMFs. Among different techniques, which can be useful to achieve characterization of blood and blood components, Raman spectroscopy is an established analytical procedure, based on the inelastically scattered light from a sample, which provides information about vibrational modes of the functional groups inside the analyzed sample. In fact, a Raman spectrum is characterized by several features, which can be specifically assigned to the chemical bonds inside the sample. Therefore, Raman technique has been successfully applied to analyze blood and blood components since the early 1970 [9]. Moreover, a burden of literature suggests that Raman spectroscopy of individual cells (performed by coupling a Raman apparatus to an optical microscope) provides meaningful biochemical information, mainly based on relative peak intensity, that permits to highlight differences between cells exposed to chemical and physical stress and control ones [10–13]. Therefore, the comparison between Raman intensity of some specific peaks of control and exposed cells can detect modifications of the cellular chemical composition, as a consequence of the exposure process. In the particular case of rounded shaped monocytes (several micrometers in diameter), wherein the nucleus occupies most of the total cellular volume, the exciting laser beam can be focused to produce a spot of the order of one micrometer diameter, thereby enabling the nuclear region of individual cells to be probed by Raman micro-spectroscopic technique.

In this study we combined optical microscopy, Raman micro-spectroscopy, high resolution-respirometry and morpho-functional confocal microscopy to investigate the biochemical/metabolic effects on leukocyte subsets from whole peripheral blood of healthy donors exposed to electromagnetic fields at 1.8 GHz (commonly employed in GSM dual band of mobile phones) for different times ranging from 1 to 20 h.

## Materials and experimental methods

### Ethics statement

Specific approval of the local ethics committee was obtained for this study (Ospedali Riuniti di Foggia–University Hospital cod. 30/CE/2014).

### Blood collection and exposure to EMF

Peripheral blood samples were collected from healthy donors in the Blood Bank of “Ospedali Riuniti” Hospital (Foggia, Italy). Healthy donors were screened through a routine laboratory assessment and then selected for blood donation. Oral informed consent was obtained from each human subject included in this study. Each blood sample from each volunteer was deposited in two Vacuette blood collection tubes (made of PET and internally coated with K_3_EDTA to avoid coagulation), inside which they were kept during exposure. The tubes were compliant with the requirements of ISO 6710 standard [14]. The EMFs-exposure protocol foresaw to hold one of the two tubes inside a home-made reverberation chamber [15, 16] at room temperature, ranging from 20°C to 23°C (variations of ± 1.5°C often occurred in the reverberation chamber, depending on the exposure time). The other tube, consisting of the control blood sample, was maintained at room temperature for the same time inside the room where the reverberation chamber was located. During exposure to EMFs in the reverberation chamber the frequency was set at 1.8 GHz and the electric field (E) strength was set at the root mean square (rms) value of 200 V/m. The exposure process was carried out at four different times: 1, 5, 12 and 20 h.

### Exposure apparatus to 1.8 GHz

For the irradiation of whole peripheral blood, we used a home-made reverberation metallic chamber at 1.8 GHz having size (150x85x85) cm^3^, designed and implemented previously [15, 16]. This metallic cavity has dimensions much larger with respect to the wavelength (λ) of the electromagnetic field at 1.8 GHz. In fact, at this frequency, λ results to be 16.6 cm and it is much smaller than the chamber dimensions. A reverberation chamber is a large multimode cavity with statistically uniform EMFs, obtained by a mechanical metallic stirrer [17]. A reverberation chamber works correctly at a frequency *f*, if the number of eigen-modes N is larger than 60 [17]. The number N is given by:

$$N=\frac{8\pi }{3}abd{\left(\frac{f}{c}\right)}^{3}-\left(a+b+d\right)\frac{f}{c}+\frac{1}{2}$$ (1)

where *a*, *b*, *d* are the sizes of the chamber and *c* is the electromagnetic wave speed in the free space.

Eq (1) is valid when the cavity dimensions are larger than a half wavelength, as in such a case. In our reverberation chamber, for *f* = 1.8 GHz, Eq (1) gives N = 1942, a value which is much larger than 60, so confirming a correct functioning of the chamber.

The internal EMF is characterized by stochastic values, but it is averagely uniform and isotropic one [18]. The transmitting antenna is a stick of 4.15 cm long, corresponding to λ/4 for the 1.8 GHz frequency and it is fixed perpendicularly to a vertical side wall of the chamber. The electromagnetic wave generator (from 100 kHz to 2112 MHz) has an output power ranging from -140 dB_m_ to +10 dB_m_ and it is connected to a first power amplifier (up to 5W in the frequency range 1.5–2.0 GHz). Another power amplifier (ranging up to 20 W for the frequency of 1.8 GHz) is connected in cascade with the previous one and its output is connected to the antenna.

The apparatus was characterized at different input RF powers by measuring the E strength in different points inside the reverberation chamber by means of an EMFs Meter (PMM 8053B) connected with an optical fiber to an Electric Field Probe (PMM EP-600) which measures the root mean square (rms) value. Its frequency range is 100 kHz—9.25 GHz. The probe was calibrated in air at the PMM Calibration Laboratory. The measurements of E strength were performed and averaged by the mentioned electric field meter over a fixed time range of 6 minutes.

The magnetic field (H) strength was also measured in the reverberation chamber by means of a magnetic field probe (HP-102) connected with an optical fiber to the EMFs meter PMM 8053B, which measures also the rms values of H. The measurements of H strength were performed in several points in the region where the blood samples were located and they were also averaged over a time range of 6 minutes, obtaining a rms value of 0.41 A/m.

To determine the SAR parameter inside the blood sample, the experimental procedure is reported in [15, 16]. Moreover, the calibration procedure performed in [5] provides a ratio E_out_/E_in_ = 15, where E_out_ is the measured E strength value averaged over 6 minutes outside the blood sample put in the reverberation chamber and E_in_ is the averaged value over 6 minutes of the E strength inside the samples. In our case, the E_in_ average value corresponding to 200 V/m is E_in_ = 200/15 V/m = 13.3 V/m. The SAR value corresponding to this E strength is given by:

$$SAR=\sigma \frac{E_{in}^{2}}{r}$$ (2)

where σ is the electrical blood conductivity and ρ is the mass density of the blood. By taking into account the blood values: σ = 1.250 S/m and ρ = 1060 Kg/m^3^ [19] and the above E_in_ value, from Eq (2) we obtain a value of SAR = 0.21 W/Kg. In the range of EMFs frequencies (from 10 MHz to 10 GHz) and, in particular, at 1.8 GHz, the SAR values of mobile devices have to be lower than 2 W/Kg for head and trunk (general public population), as predicted by the guidelines on exposure limit to EMFs ruled by the International Commission on Non Ionizing Radiation Protection (ICNIRP) [20, 21]. For the European countries, at 1.8 GHz the permitted SAR values of the mobile phones are lower than the previous one and they are 0.3 W/Kg around the human head and 0.5 W/Kg for the human body measured at 1.5 cm far away [22]. Therefore, the SAR value achieved in our apparatus is of the same order of magnitude of the maximum allowed value in the European countries.

### Isolation of PBLM cells and optical microscopy analysis

Peripheral blood lympho-monocyte (PBLM) cells were obtained from the peripheral blood samples after Ficoll-Hypaque density gradient separation. The lympho-monocyte cells obtained from the buffy coat were seeded in 6-well plates wherein poly-lysine coated coverslips had been placed in complete RPMI 1640 medium supplemented with 10% fetal bovine serum (FBS). Mainly monocytes attach to the covers after 4h of incubation while most of lymphocytes remain suspended in the liquid portion. The liquid portion was aspirated and the coverslips with adherent PBLM were recovered. Cells grown on coverslips were fixed with 3.7% para-formaldehyde (PFA) in phosphate buffer saline (PBS). Morphological observations were also performed by staining cells with the May-Grunwald-Giemsa method and observing the samples by means of an optical microscope (Olympus IX71) operating in bright-field mode.

### Raman measurements and processing

For Raman microspectroscopy measurements, a glass coverslip with adherent PBLM cells was placed on a microscope slide provided with a well, which was filled with PBS solution. The face of the coverslip where the cells adhered was placed in the PBS solution so that the laser beam through the glass coverslip probed fixed single cells kept in the PBS solution [23]. We checked that this configuration did not lead to the degradation of the samples as a result of photochemical reactions or sample heating during the measurement time.

Raman spectra were recorded at room temperature by means of a Raman confocal microspectrometer apparatus (Labram from Jobin-Yvon Horiba). The exciting beam, consisting of the 488 nm line of an Ar ion laser, was focused on the sample by using an Olympus optical microscope with a 100x oil-immersion objective (1.3 numerical aperture), resulting in a diffraction-limited spot about 2 μm in diameter. The laser power on the sample was properly fixed at 5 mW (corresponding to a laser intensity of about 1.5x10^5^ W/cm^2^) to obtain a good signal/noise ratio while avoiding thermal damage of the sample. The reproducibility of the spectra using such excitation intensity was verified by measuring three spectra in sequence on the same sample point. Each Raman spectrum was measured in the 700–1800 cm^-1^ spectral range, where information about the functional groups of the main cellular components (proteins, nucleic acids, lipids, and carbohydrates) are included. The acquisition time was set at 20 s for each single spectral acquisition and the signal was averaged for three acquisitions. The light scattered from the sample was collected using the same 100x oil-immersion objective (in backscattering geometry) and passed through a notch filter (Kaiser Optical Systems, Inc.) to suppress the laser line. The Raman scattered light crossed a square confocal hole (500 μm diagonal) and a 200 μm entrance slit of a spectrometer equipped with a 600 grooves/mm grating. The signal was detected by means of a charge-coupled device (CCD) cooled at 223 K. A separate CCD camera was used to record white-light microscope images of the cells to be probed. The spectral resolution was ~5 cm^-1^/pixel. Raman spectra were recorded from many single cells, randomly chosen on each glass coverslip containing the control cells and the cells exposed to the EMFs. Raman spectra were measured by focusing the laser spot on the cell surface above the nucleus in order to reduce the cell-to-cell variability and to obtain information originating from the plasmatic membrane, nucleus, and cytoplasmatic region between the plasmatic membrane and nucleus. Raman spectra of the background signal (the coated glass coverslip and the PBS solution) were acquired after the acquisition of each cell spectrum by moving the objective, without varying the focus position, to a close region where no cells were located.

Raman spectrum collected from each single cell was preprocessed firstly by subtracting the background signal from each spectrum; then, by performing a subtraction of the cell fluorescence and stray light signal, described by a fifth-order polynomial function; finally, by dividing the intensity of each wavenumber channel of the Raman spectrum by the total spectral intensity, to minimize the effect of variation either in the sampling of the cellular volume or in the laser power on the spectral intensity of each single cell (this last procedure normalizes to the total amount of biological material in the sampling volume). After this pretreatment procedure, the normalized spectra collected from each coverslip were independently averaged to obtain an average spectrum for both the unexposed and exposed cells.

### Respirometric analysis

Isolated PBLM cells were isolated as described above, washed in PBS, resuspended in RPMI 1640 medium at about 10^7^ cells/ml and immediately assayed for O_2_ consumption rates (OCR) by high resolution oxymetry (Oxygraph-2k, Oroboros Instruments) at 37°C under continuous stirring. After attainment of a stationary resting endogenous substrate-sustained respiratory rate (OCRr), 1 μg/ml of oligomycin was added and the resulting respiratory activity (OCRo) recorded for 5 min. Finally, 1 μg/ml of valinomycin was added to obtain the uncoupled respiration (OCRu). All the OCRs were corrected for 2 μM rotenone-insensitive respiration and normalized to the cell number. The respiratory control ratio (RCR) was obtained as OCRr/OCRo; the respiratory activity linked to the activity of the FoF1-ATP synthase was obtained as OCRr-OCRo and referred as OCR_ATP_. To note, frozen PBLM cells samples were unsuited for this analysis because after thawing ensued non-reproducible results.

### Confocal microscopy analysis

Laser scanning confocal microscopy (LSCM) live cell imaging was carried out as in [24]. Briefly, buffy coat-derived PBLM cells were seeded on Cell Tak^TM^-coated 35-mm glass-bottom dishes and incubated for 20–30 min at 37°C either with 0.1 μM tetramethylrhodamine ethyl ester (TMRE) to detect ΔΨ_m_ or with 0.5 μM MitoTracker^®^ Green FM to detect mitochondria or with 10 μM 2,7-dichlorofluorescin diacetate, which is converted to dichlorofluorescein (DCF) by intracellular esterases, to detect ROS. Stained cells were washed with PBS and examined with a Leica TCS SP8 confocal laser scanning microscope (images collected using a 60× objective [1.4 NA]); TMRE red fluorescence was elicited with the He–Ne laser beam (λ_ex_ 543 nm), MitoTracker and DCF green fluorescence was elicited with the Ar–Kr laser beam (λ_ex_ 488 nm). Acquisition, storage, and analysis of data were performed with a dedicated instrumental software from Leica or ImageJ version 1.37 (https://imagej.nih.gov/ij/).

### Measurements of citrate synthase and cytochrome c oxidase activity

The activity of mitochondrial marker enzymes was assayed spectrophotometrically on total PBLM cell lysates as in [25]. Briefly, 5-10x10^6^ cells were suspended in 0.5 ml of 0.32 M sucrose, 40 mM KCl, 20 mM Tris-HCl (pH 7.2) 2 mM EGTA and subjected to ultrasound treatment till complete rupture of the cell membranes (assessed by trypan blue assay). PBLM lisates were diluted 1:8 in 10 mM Tris-HCl (pH 8.0), 1mg/ml BSA and cytochrome c oxidase (complex IV) was assayed (in the presence of 2.0 μM antymicin A plus 1.0 μM rotenone) following the initial 2 mM KCN-sensitive rate of 20 μm ferro-cytochrome *c* oxidation under aerobic conditions (*ɛ*_550nm_ = 19.1 mM^−1^ cm^−1^). Ferro-cytochrome c was attained treating 1–2 mM ferri-cytochrome *c* (in dd-water) with a few grains of sodium dithionite and dialyzed overnight against dd-water. Citrate synthase activity was assayed spectrophotometrically in Tris-HCl (pH 8.0) measuring the absorbance increase of the reaction between 0.3 mM Acetyl-CoA and 0.5 mM oxaloacetate coupled with the reaction of the released CoA-SH with 0.1 mM DTNB (5,5′-dithiobis (2-nitrobenzoic acid)) to form 5-thio-2-nitrobenzoic acid (TNB) (*ɛ*_412nm_ = 13.6 mm^−1^ cm^−1^). The activities of both cytochrome c oxidase and citrate synthase were normalized to the initial cell number.

### Statistical analysis

Data are presented as means ± S.E.M. and analyzed by two-tailed student t-test using SigmaPlot version 9.0 (Systat Software). A value of p < 0.05 was set as statistically significant and further validated by the variance analysis test (ANOVA) followed by a post hoc Bonferroni test.

## Results and discussion

### Morphological analysis of the adherent component of PBLM

After exposure of whole blood to EMFs the PBLM cells were isolated, seeded on polylysine-coated dishes and visualized by optical microscopy. Under this condition the mononocyte fraction of PBLM, along with platelets, adhere to the conditioned plastic surface while lymphocytes largely remain in the surnatant.

**Fig 1:**
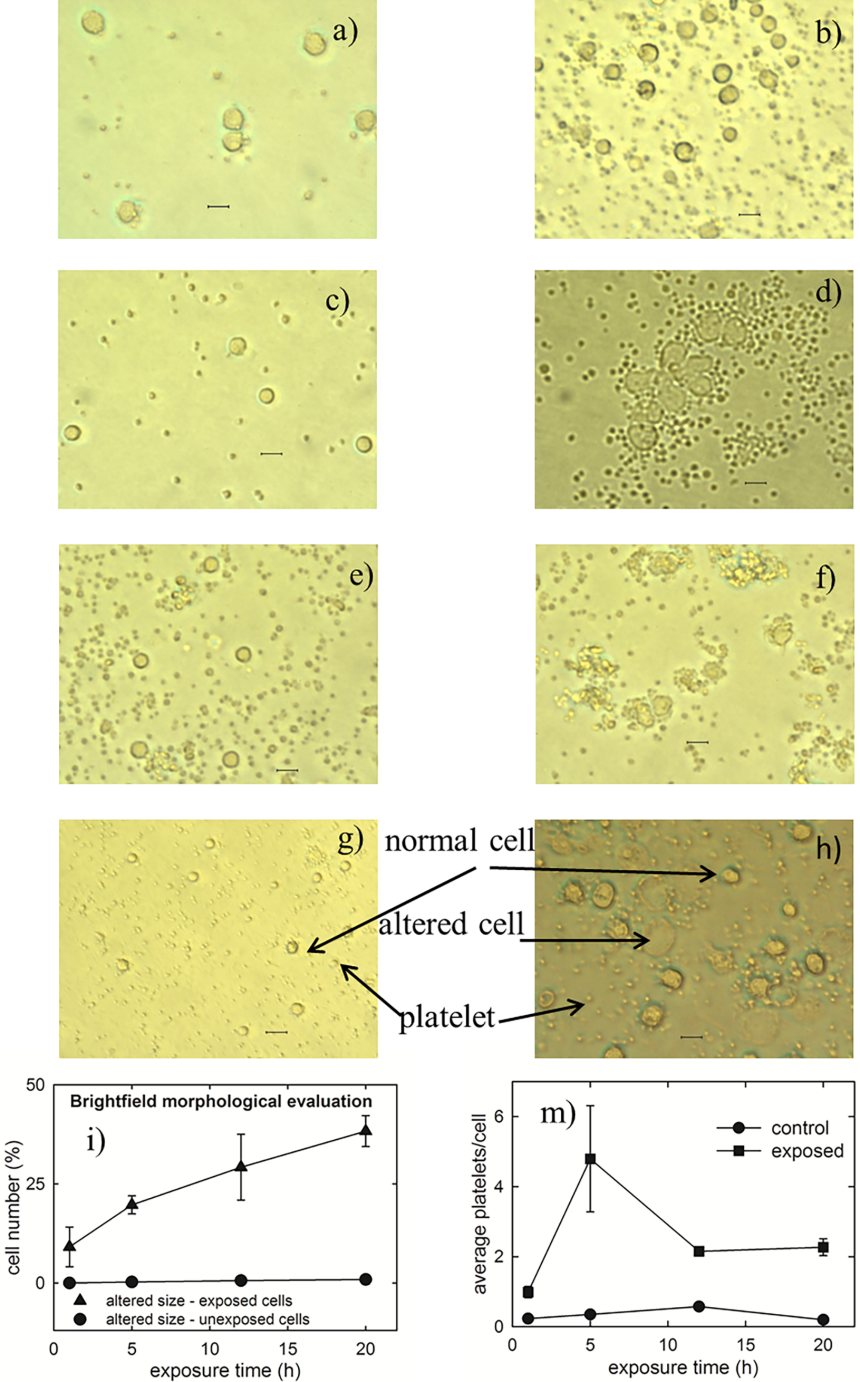
Optical images of control and exposed cells to EMFs at 1.8 GHz. Bright field optical images of controls (a, c, e, g) and respective 1 h, 5 h, 12 h and 20 h exposed (b, d, f, h) samples to the electric field strength of 200 V/m of microwave of 1.8 Ghz. The larger structures correspond to adherent PBLM cells, whereas the smaller ones are platelets which cannot be completely separated by the density gradient procedure to isolate PBLM cells. The scale bar corresponds to 15 μm. The images are representative of 3–4 independent experiments (i.e. biological replicates corresponding to samples from different donors for each condition), yielding comparable results. The plots at the bottom shows the percentage of cells whose size appeared altered after 1, 5, 12 and 20 h exposure (i) and the average number of platelets stuck on each single cell as a function of exposure time (m). For each sample, about 300 cells were analyzed from 20–30 randomly selected optical fields. The bar values are means ± SEM of 3–4 independent experiments.

At the E strength of 200 V/m, no clear difference in the size and shape of cells after 1 h exposure can be detected, as can be seen comparing Fig 1A (control cells) and Fig 1B (exposed cells). Instead, some morphological effects in the PBLM cells can be observed after 5 h exposure: in fact, a few cells seem to aggregate each other after this time (compare the control sample in Fig 1C with the 5 h exposed sample in Fig 1D). In addition a small increase in the average size of a few cells can be observed. Such increased-size cells are also present in the sample exposed for 12 h, as can be seen by comparing Fig 1E (control sample) with Fig 1F (12 h exposed sample). In this case the aggregation effect is less evident with respect to the sample exposed for 5 h. Following 20 h of EMF exposure quite large morphological modifications occurred in adherent cells as compared with non-exposed samples (compare Fig 1G and 1H) consisting in an enlarged and flattened surface. The percentage of morphologically altered cells (i.e. characterized by a larger size and/or deformed shape with respect to those of typical control cells or showing an aggregation status) has been estimated by analyzing many optical fields of exposed samples and the results are reported in Fig 1I. It is shown that the percentage of morphologically altered cells increases with the exposure time (triangles), whereas it is negligible and time-independent in the control samples (circles). By a careful inspection of optical images, it is also evident the trend of platelets in exposed samples to cluster each other and to stick to the cells. Such a behavior is particularly evident after 5 h of exposure, but is it is also visible in the 12 h and 20 h exposed samples, whereas it is less evident after 1 h exposure, although the average number of platelets stuck to each single cells in exposed samples results larger than the corresponding number in control samples, as visible in Fig 1M.

**Fig 2:**
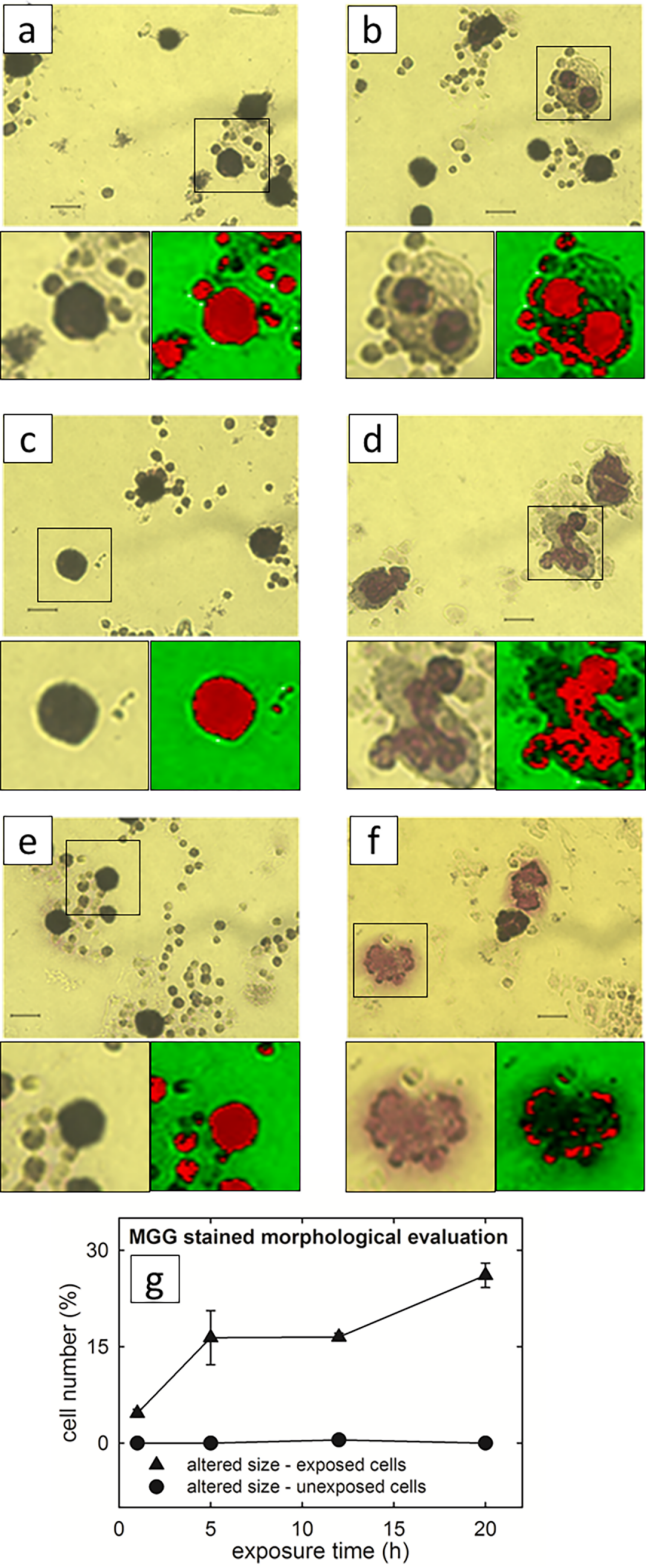
Optical images of stained control and exposed lympho-monocyte cells to EMFs at 1.8 GHz. Representative May-Grunwald-Giemsa (MGG) images of controls (a, c, e) and respective 5 h, 12 h and 20 h exposed (b, d, f) samples to the electric field strength of 200 V/m of microwave at 1.8 GHz. The scale bar corresponds to 7 μm. Digital magnifications of selected cells are shown along with their rendering obtained by the software ImageJ 1.49 (http://imagej.nih.gov/ij) with the tool LUT-ICA. The plot g) shows the percentage of MGG stained cells whose size appeared altered after 1, 5, 12 and 20 h exposure. For each sample, about 300 cells were analyzed from 20–30 randomly selected optical fields. The bar values are means ± SEM. The images are representative of 3–4 independent experiments (i.e. biological replicates corresponding to samples from different donors for each condition), yielding comparable results.

In Fig 2 the adherent cell samples have been stained with May-Grunwald-Giemsa method to better appreciate the differences between control cells and exposed ones, especially about the nucleus region, which results more intensely stained because of the molecular interaction between eosin Y and an azure B-DNA complex. Such differences are quite similar to those described in Fig 1. Also for this test the percentage of cells characterized by anomalous cell and nucleus size/shape has been estimated in the exposed cells and compared with the control ones. As shown in the plot of Fig 2I, the morphological changes for exposed samples increase with increasing exposure time, whereas the corresponding control samples show a negligible number of altered size cells. A closer image analysis of selected cells, displaying altered size and morphology, revealed clear alterations in the nuclear chromatin staining documenting transitions from a compact circular shape in the controls (Fig 2A, 2C and 2E) to irregular multi-lobate/fragmented structures upon EMF-exposure (Fig 2B, 2D and 2F). The observed morphological changes can be indicative of biochemical modifications occurring in PBLM exposed to EMFs [26] or of altered adhesiveness of a different cell phenotype present in the PBLM fraction. Evident morphological effects induced by EMFs exposure to 1.8 GHz and at a SAR value of 1.25 W/Kg were also observed by Ashraf et al. [27]. Similarly to what observed in Fig 1 also the stained platelet fraction of PBLM appear to have a tendency to form aggregation among them and with PBLM.

### Raman spectroscopy analysis of the adherent component of PBLM

**Fig 3:**
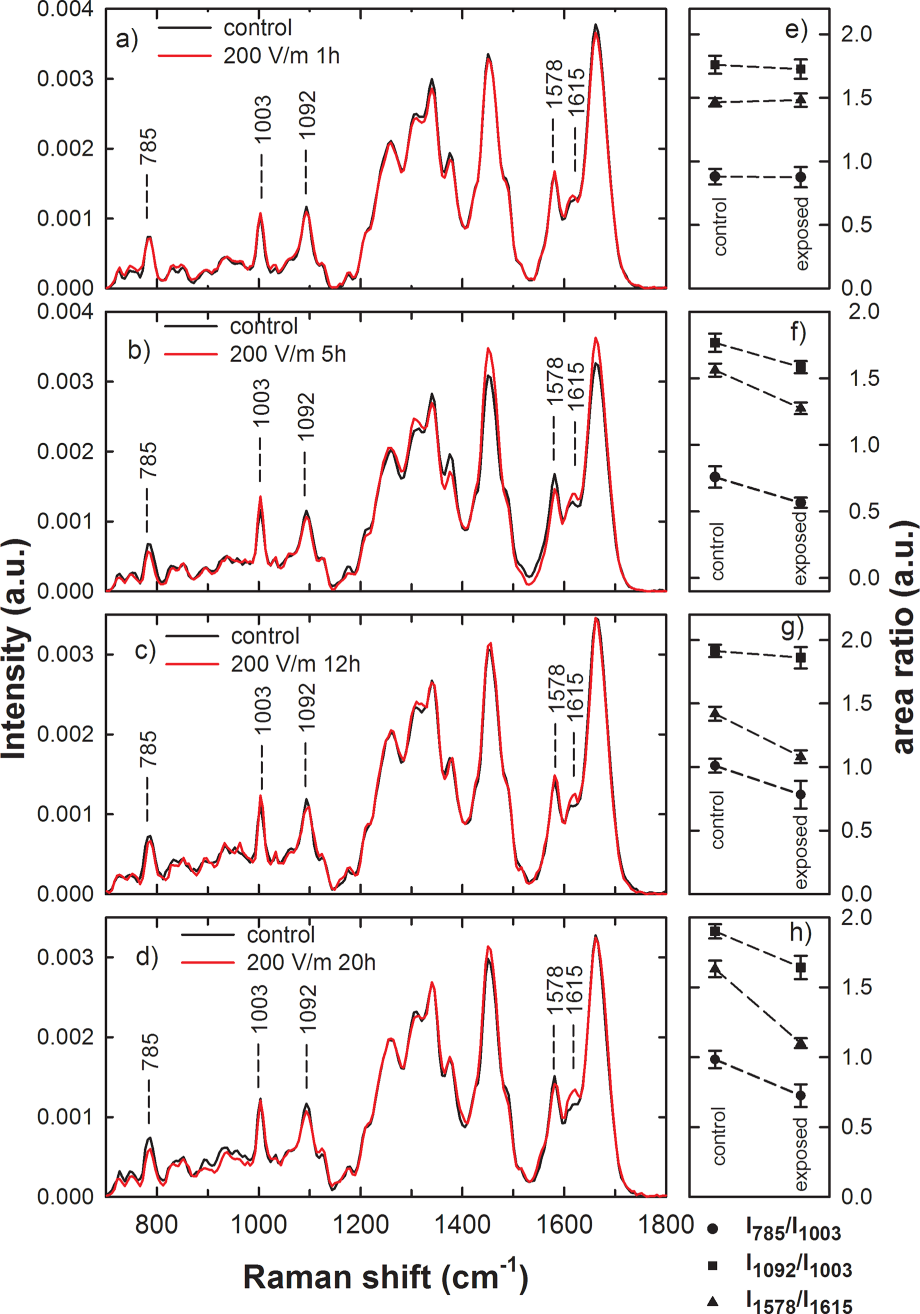
Average Raman spectra of control and exposed PBLM cells to EMFs at 1.8 GHz. Raman spectra of control (continuous black line) and exposed (continuous red line) PBLM cells which were extracted from whole blood samples. The irradiated PBLM cells were exposed to the electric field strength of 200 V/m of microwave at 1.8 GHz inside a reverberation chamber for 1 (a), 5 (b), 12 (c) and 20 (d) h. The labeled Raman features are related to DNA (at 785, 1092 and 1578 cm^-1^) and protein (at 1003 and 1615 cm^-1^) content (see text). Each spectra is the average from 20 different cells under each condition and are representative of 3–4 independent experiments (i.e. biological replicates corresponding to samples from different donors for each condition). On the right hand side of each Raman spectrum is shown the corresponding comparison between mean values of the I_785_/I_1003_ (dots), I_1092_/I_1003_ (squares) and I_1578_/I_1615_ (triangles) intensity ratios of Raman peaks of control and 1 (e), 5 (f), 12 (g) and 20 (h) h EMFs exposed cells. The values are means ± SEM of 3–4 independent experiments (i.e. biological replicates corresponding to samples from different donors for each condition).

Raman spectra have been collected from control and exposed adherent PBLM cells, whose shape and size were not deeply altered by EMFs exposure, in order to investigate the presence of earlier markers of biochemical alterations in addition to or anticipating the observed morphological changes. Unfortunately, the flatness of the deformed cells shown in Figs 1 and 2 made difficult to focalize the laser beam in an intracellular volume, thus leading to unreliable spectra. The averaged Raman spectra of normalized signals from control and exposed cells derived from the same patient are shown in Fig 3 for exposure time of 1 (a), 5 (b), 12 (c) and 20 (d) h at 200 V/m. Peak positions and assignments, according to literature [28], are reported in Table 1. As shown in Fig 3, the spectra of control and exposed cells are similar for each investigated exposure time, although the relative intensity of some spectral features shows some differences in all the average spectra. Such peaks, labeled in Fig 3, are specifically related to: the ring stretching mode of DNA bases and the O-P-O stretching mode of phosphodiester bond of phosphate group of DNA (785 cm^-1^); the breathing mode of phenylalanine (1003 cm^-1^); the PO_2_^-^ phosphodioxy bond of phosphate group of DNA (1092 cm^-1^), which can be considered a marker mode for DNA (although such peak involves also the contribution from C-N stretching mode of proteins and lipids); the ring breathing mode of DNA bases (1578 cm^-1^); the C = C stretching mode of phenylalanine, tyrosine and tryptophan (1615 cm^-1^).

**Table 1:** Attribution of Raman peaks.

Spectral range of Raman features (cm^-1^) [28]	Present work (cm^-1^)	Assignment
720–725	725	(ring breathing, n.a.)
746–748	747	(ring breathing, n.a.)
780–788	785	(O-P-O str,. n.a.)+(ring breathing, n.a.)
810–859	830, 853	(O-P-O str,. n.a.)+(ring breathing, Tyr.)
	893, 932, 964	vibrational modes of glass substrate
1000–1006	1003	(symmetric ring breathing, Phe)
1031–1033	1031	(C-H in-plane bend., Phe)
1053–1064	1060	(C-O C-C, c.)+ (C-N str., p.) +(C-C str., l.)
1087–1096	1092	(PO_2_^-^ str., n. a.)
1123–1128	1127	(C-N str., p.)+ (C-O, c.) +(C-C str., l.)
1163–1176	1173	(C-H bending, Tyr)
1220–1284	1223, 1260	(Amide III, p.)
1300–1313	1307	(CH_2_ twist., l.)
1335–1343	1341	(CH_3_ def., CH_2_ wagg., p. and n.a.)
1360–1379	1378	(CH bend., Trp)+(ring breathing, n.a.) +(CH_3_ bend., l.)
1420–1421	1416	(ring breathing, n.a.)
1436–1460	1460	(CH_2_ sciss., l.)+ (CH_2_ sciss., p.)
1485–1490	1488	(ring breathing, n.a.)
1573–1582	1578	(ring breathing, n.a.)
1615–1618	1615	(C = C, Tyr., Trp.)
1655–1685	1665	(Amide I, p.) + (C = C str., l.)

Assignment of Raman spectral structures, based on the results reported in literature [28] and in the present investigation. Abbreviations: Phe: phenylalanine, Tyr: tyrosine, Trp: tryptophan, p: proteins, c: carbohydrates, l: lipids, n.a.: nucleic acids, str: stretching, bend: bending, wagg: wagging, twist: twisting, sciss.: scissoring.

The change of intensity of the above-reported spectral peaks after EMFs exposure can be related to local modification of DNA and proteins bonds within the cells. Consequently, the comparison of the intensity of these peaks in control and exposed cells can be related to modifications in cellular components as a consequence of the EMFs action. In particular, it is evident in Fig 3 the decrease of the signals related to DNA structure at 785 and 1092 cm^-1^ after exposure to the EMFs for exposure time longer than 1 h, so suggesting some subtle change in the DNA dynamic structure induced by the EMFs when the exposure time is sufficiently long. Conversely, the signal related to aromatic amino-acids vibrational modes at 1615 cm^-1^ seems to increase when exposure occurs.

A proper way to get reliable biochemical information by Raman spectra is to perform ratiometric analysis, i.e. to consider the ratio between the area (or intensity) of a peak with respect to that of another one. Such a procedure also softens the artefact arising from signal fluctuations due to instrumental parameters variability, to sample and to substrate effects [29]. Therefore, the Raman peaks have been analyzed in detail, after deconvolution of each measured spectrum by means of Voigt functions, in order to highlight spectral differences. Such fitting analysis has been performed in the spectral regions between 700 and 1150 cm^-1^ and between 1530 and 1750 cm^-1^, because such regions include signals accurately attributed to specific cellular components. In particular, we compared the ratio of the area of the 785 cm^-1^ peak with respect to that of the 1003 cm^-1^ peak (I_785_/I_1003_) as a marker of the DNA-related modification with respect to that of proteins, similarly to the ratio of the area of 1092 cm^-1^ peak with respect to that of the 1003 cm^-1^ peak (I_1092_/I_1003_). In addition, we also analyzed the ratio of the area of 1578 cm^-1^ peak with respect to that of the 1615 cm^-1^ peak (I_1578_/I_1615_) which also reflects the ratio between the vibrational modes of the C = C bonds in nucleic acid and protein [30]. The spectral region between 1150 and 1530 cm^-1^ contains also some features specific of nucleic acid components (at about 1420 and 1485 cm^-1^) but they are scarcely resolved and overwhelmed by the large and intense band at about 1450 cm^-1^, which is due to both protein and lipid components. Therefore, such spectral region has been neglected for the fitting analysis.

Fig 3 (panels on the right hand side of the corresponding averaged Raman spectra) shows the mean values of the three above mentioned area ratios for control and exposed samples. The t-test, performed to quantify the level of statistically significant difference between the two groups of area ratio in control and exposed PBLM spectra, yields the values reported in Table 2. It can be inferred that the difference in the mean values of the two groups is large enough to reject the possibility that the difference is due to random sampling variability for all the intensity ratios related to 5, 12 and 20 h exposure, except for the comparison between the I_1092_/I_1003_ after 12 h exposure (Fig 3F, 3G and 3H, respectively and the p-values reported in Table 2). On the contrary, there is no statistically significant difference between the area ratio groups related to 1 h exposure (Fig 3E and the p-values reported in Table 2). The Raman spectral change in the ratio between DNA and protein components, attained at EMFs exposure longer than 5 h, is mainly due to decrease of the signal related to the former with respect to that of the latter. Both chemical bonds inside single nucleic acids as well as among nucleic acids appear to be modified by EMFs exposure. We retain that the EMFs cause a chemical modification mainly in the environment surrounding the nucleus district. Consequently, the signal of the Raman peaks related to bonds close to such modified environment is the most affected by the exposure procedure. Nonetheless, the number of bonds that are affected after exposure is relatively low (see the difference of the above mentioned signals in control and exposed samples in Fig 3), so that a shift of the corresponding Raman peaks is not clearly visible in the spectra.

**Table 2:** t-test of area ratios of Raman peaks in exposed with respect to control samples.

*Exposure time*	I_785_/I_1003_	I_1092_/I_1003_	I_1578_/I_10615_
20 h	0.0142	0.0094	5.4∙10^−9^
12 h	0.0431	**0.560**	4∙10^−4^
5 h	0.0334	0.0299	7∙10^−5^
1 h	**0.9645**	**0.7403**	**0.7795**

p-values of the t-test performed between the I_785_/I_1003_, I_1092_/I_1003_ and I_1578_/I_1615_ area ratios of Raman peaks of Fig 3 for the control and exposed lympho-monocyte samples to EMFs of 1.8 Ghz. The above ratios have been calculated by considering about 20 Raman spectra of control and exposed cells. The p-values which do not correspond to a statistically significant difference between the two groups, by considering a significance threshold of p < 0.05, are reported in bold.

**Fig 4:**
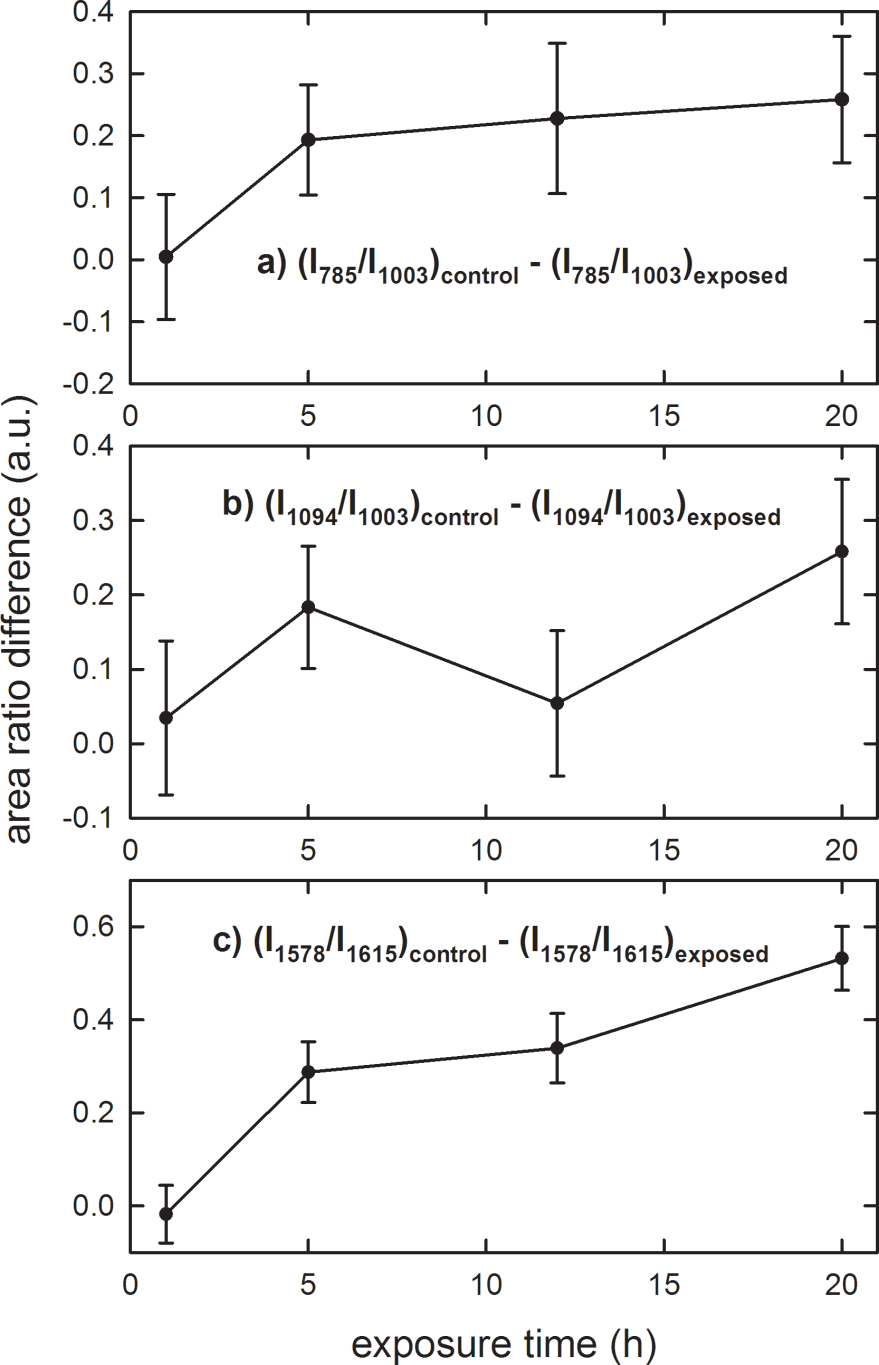
Area ratios of Raman peaks from spectra of control and exposed PBLM cells to EMFs at 1.8 GHz. Difference of I_785_/I_1003_ (a), I_1092_/I_1003_ (b) and I_1578_/I_1615_ (c) area ratios of Raman peaks of the control with respect to the exposed lympho-monocyte samples for different exposure time of Fig 3. The values are means ± SEM of 3–4 independent experiments (i.e. biological replicates corresponding to samples from different donors for each condition).

In order to estimate the exposure time dependence of the chemical modifications inside DNA components, Fig 4 shows the difference of investigated area ratios of the control with respect to the exposed PBLM samples for different exposure times. It is clearly evident that such a difference is negligible for 1 h exposure but results positive for the longer exposure times, so confirming that the three investigated area ratio values are larger for the control than for the exposed samples. It is interesting to remark that the intensity ratio difference is characterized by an increasing trend versus the exposure time. In keeping that the energy transfer occurring at the used EMF exposure is not sufficient to cause breaking of covalent bonds within biomolecules, it is likely that the observed changes in the Raman signals, rather reflect localized alterations in the sovramolecular geometry of the DNA double-helix assembly. Consistently, denaturation of DNA was reported to be accompanied with a decrease of the Raman signals assigned to the phosphodiester bond moiety [31, 32].

### Effect of EMF exposure on the mitochondrial respiratory activity in PBLM cells

**Fig 5:**
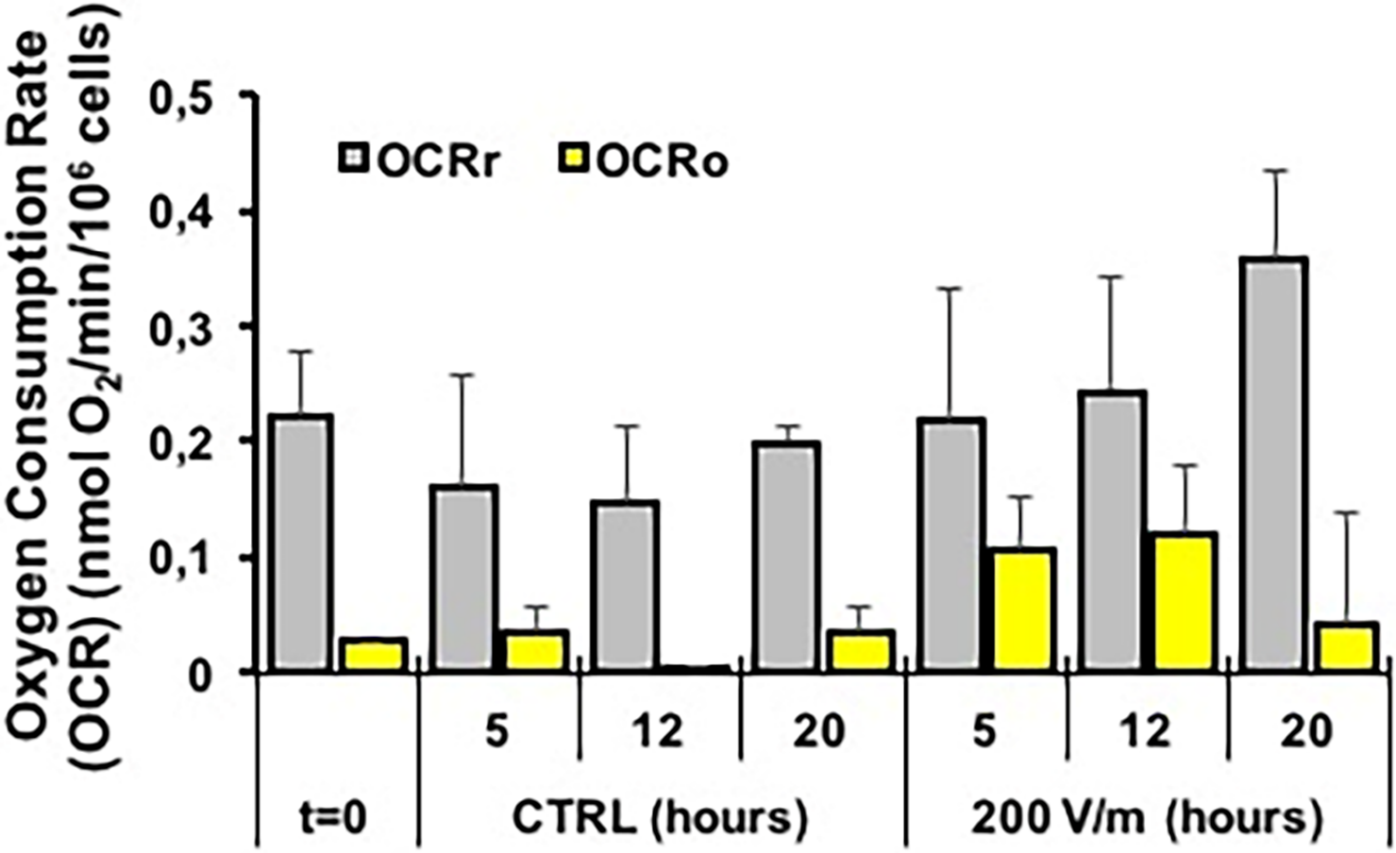
Mitochondrial respiratory analysis in intact PBLM cells. PBLM cells isolated from human subjects were tested for oxygen consumption rates (OCRs) by high resolution respirometry as described under Materials and Methods. Respiratory activity expressed as nmol O_2_/min/10^6^ PBLM cells. OCRr and OCRo are activities attained before and after addition of oligomycin and were corrected for rotenone-insensitive respiration. The bar values are means ± SEM of 3 independent experiment under each condition; t = 0, activity measured in freshly isolated PBLM cells; PBLM cells incubated for the indicated h in the absence (CTRL) or in the presence of EF (strength of 200 V/m).

Finally, we sought to investigate if exposure to EMFs caused alterations in cell metabolism. Given the central role played in this context by the mitochondrial OxPhos as a major ATP producer we carried out a systematic analysis using high-resolution oxymetric methodology. Fig 5 shows comparatively the oxygen consumption rates (OCRs) measured in intact PBLM cells treated for the indicated times at the E strength of 200 V/m. PBLM cells isolated from the same human subject were incubated under identical conditions and served as internal control (CTRL). The OCRs were corrected for the rotenone-insensitive respiratory activity. Since rotenone is a specific inhibitor of the NADH-dehydrogenase (Complex I of the mitochondrial respiratory chain) all the reported OCRs can be ascribed to the mitochondrial respiratory activity. The OCRr (gray bars) measured under resting condition (i.e. utilizing internal or medium-contained substrates) is shown. No significant differences were observed in not-EMFs-exposed PBLM cells at 5, 12 and 20 h of incubation as compared with samples tested soon after their isolation. Five h of EMFs-exposure resulted in no difference as referred to the cognate control. At 12 and 20 h of EMFs-exposure, a trend toward a progressive increase in the OCRr was observed. However, the averaged values did not reach statistical significance likely because of the large inter-individual variability.

**Fig 6:**
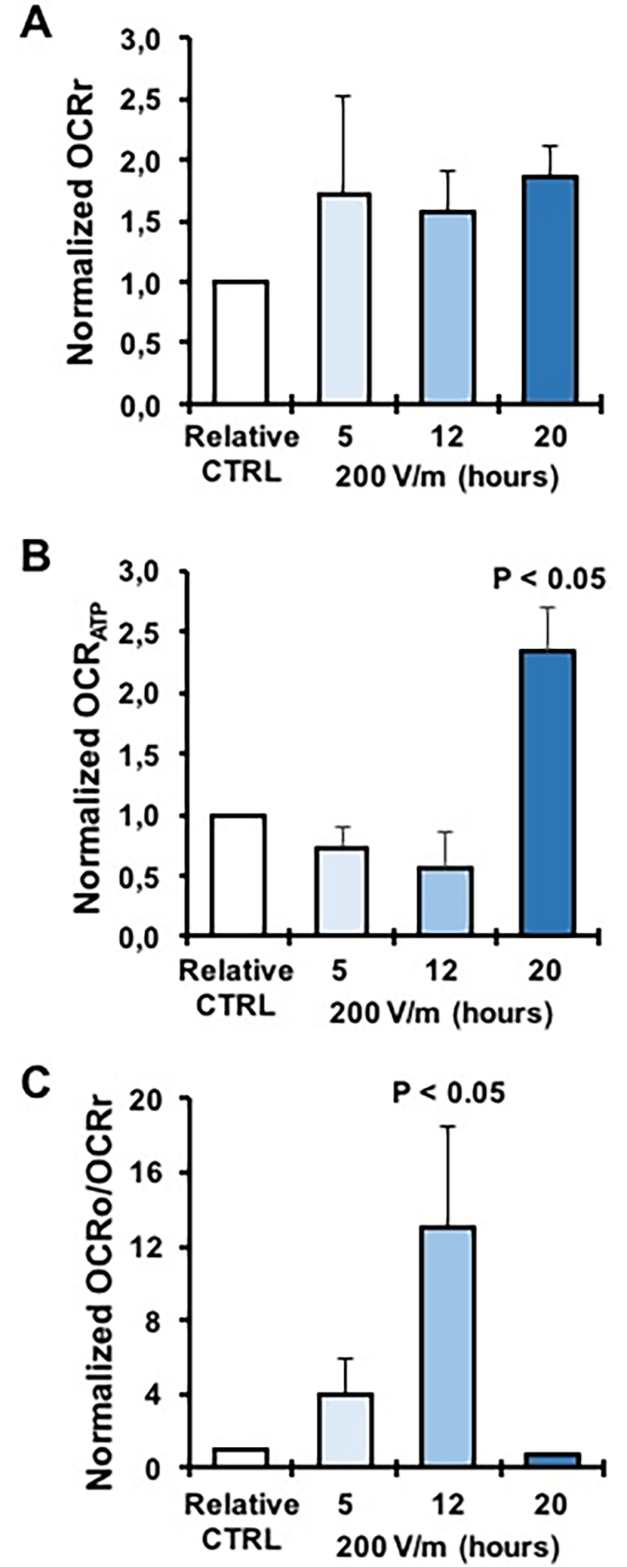
Mitochondrial respiratory analysis in intact PBLM cells. (**A**) OCRr of EMFs-exposed PBMN cells normalized to the untreated cognate control samples incubated for the same time and under identical conditions. The bar values are means ± SEM of 3 independent experiment under each condition and were calculated from the absolute values shown as averages in Fig 5. (**B**) OCR_ATP_ of EMFs-exposed PBLM cells normalized to the untreated cognate control samples incubated for the same time and under identical conditions. The bar values are means ± SEM of 3 independent experiment under each condition and were calculated as OCRr-OCRo from the absolute values shown as averages in Fig 5. (C) OCRo was divided by the OCRr of the same assay and normalized to the relative CTRL value. The bar values are means ± SEM of 3 independent experiment under each condition and were calculated as from the absolute values shown as averages in Fig 5.

Normalization to the internal control of the same patient’s sample resulted in a significant 50–60% increase at 12 and 20 h of EMFs exposure (Fig 6A). Addition of the FoF1 ATP synthase inhibitor oligomycin caused a decrease of the OCR (Fig 5, yellow bars). This is expected since inhibition of the FoF1 ATP synthase blocks the utilization of the proton gradient generated by the respiratory chain that inducing a rise in the mitochondrial transmembrane potential (ΔΨ_m_) in turn back-inhibits the electron transfer in the respiratory chain [33]. Indeed, addition of a protonophore uncoupler (FCCP) or of a K^+^ ionophore (valinomycin) dissipating the ΔΨ_m_ re-established the OCR to the resting values. The difference OCRr-OCRo (OCR_ATP_) is taken as an indirect measure of the oxygen consumption linked to the ATP synthesis [33]. Fig 6B shows that the normalized OCR_ATP_ decreased at 5 and 12 h of exposure while increased at 20 h of EMFs exposure. The absolute value of the residual OCRo is considered an indication of the proton back-flow (leak) caused by the passive proton permeability of the mitochondrial membrane and/or by membrane-bound uncoupling proteins (UCPs, PTP) [33]. Figs 5 and 6C show that the OCRo either as absolute value or when normalized to the OCRr respectively increased at 5 and 12 h of EMFs exposure while decreased at 20 h of EMFs. All together the presented results suggest that following EMFs exposure PBLM cells undergo progressive changes in their oxidative metabolism. Relatively short time of exposure to EMFs (5 to 12 h in our experimental protocol) caused impairment in the coupling efficiency of the mitochondrial OxPhos system which appear to be mainly due to enhanced dissipative flow of protons at the level of the inner mitochondrial membrane. Theoretical considerations have envisaged the possibility that irradiated electromagnetic fields might interfere with the localized electric field generated at the level of the mitochondrial inner membrane by the proton-motive activity of the respiratory chain (120–180 mV negative inside) [34, 35]. Longer exposure to EMFs (20 h) puts in action compensatory mechanisms to counteract the harmful dysfunctioning of mitochondria. Though the mechanism of both the effect of EMFs and the following resilient adaptation in PBLM cells remains to be elucidated its impact on the biology of immune-competent cells warrants consideration. The metabolism in immune-competent cells is emerging as an important factor controlling both the innate and acquired immune response and activation of immune-competent cells is accompanied with rewiring of the metabolic phenotype [36]. In particular, lymphocytes and monocytes proved to rely on OxPhos metabolism in their inactive/quiescent state and to shift toward aerobic glycolysis once activated by pathogen invasion [37]. The presented results imply concerns about the risk deriving from long-time exposure to EMFs. Indeed, if immune competent cells are primed to up-regulate their oxidative metabolism as a general adaptive response to initial EMF insult, it is feasible to envisage that their immunometabolic shift toward a glycolysis-competent phenotype might be delayed or even hampered. Consistently, a number of studies reported deregulation of the immune response under EFMs-induced stressing conditions [38].

### Confocal microscopy analysis of mitochondria-related parameters in EMFs-exposed PBLM cells

**Fig 7:**
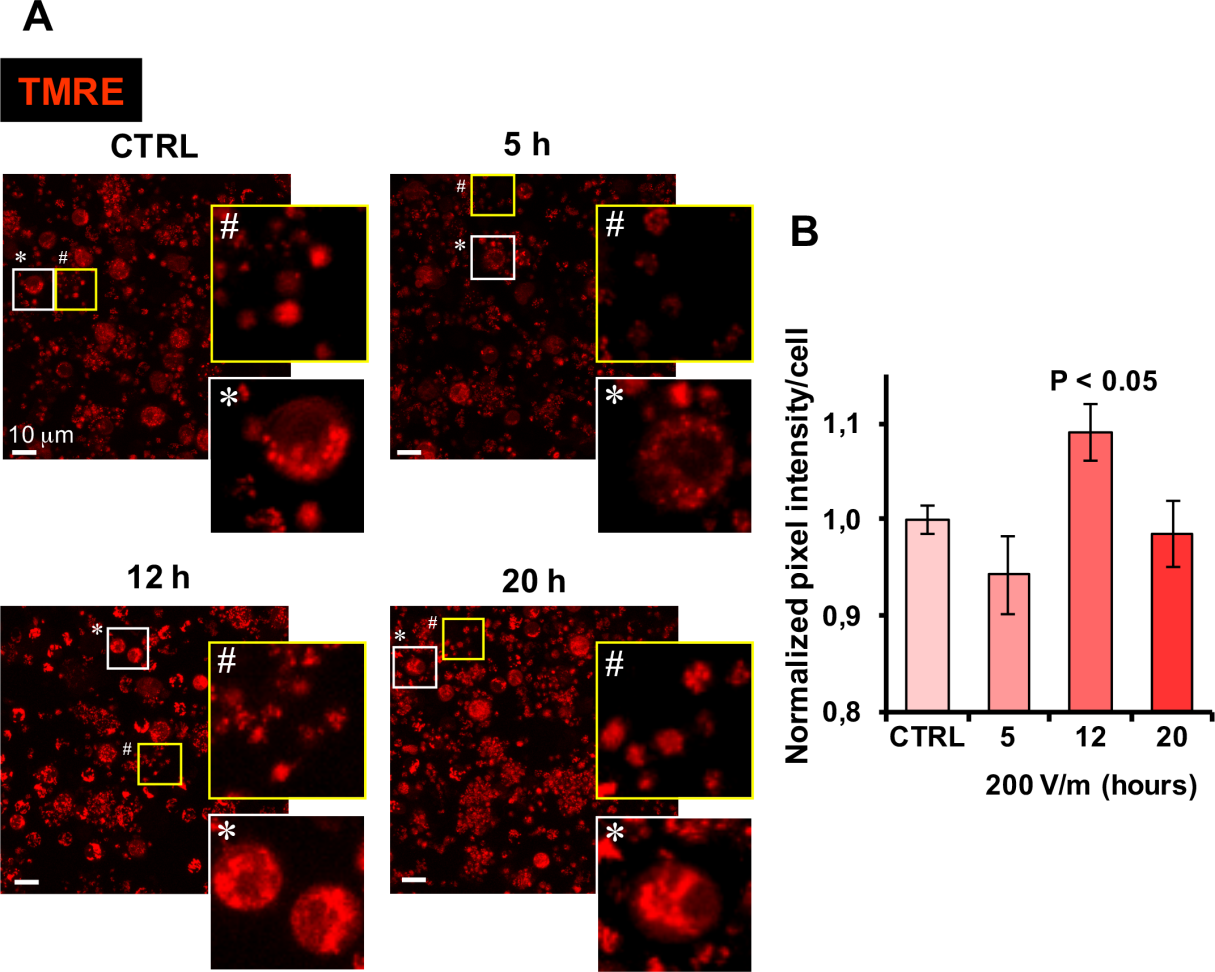
Confocal microscopy analysis of ΔΨ_m_ in EMFs-exposed PBLM cells. PBLM cells exposed to EMFs for the indicated time were incubated with TMRE and analyzed by confocal microscopy as detailed in Materials and Methods. (**A**) Representative images are shown with enlargements from each optical fields of selected nucleated cells and platelets. (**B**) Quantitative analysis of the TMRE-related fluorescence intensity/cell. The values are means ±SEM of 3 independent biological replicates; for each biological replicate the digitalized mean fluorescence/cell (ranging from 0 to 255) was averaged from 50 to 100 single cell analysis from at least three different optical fields for each condition. Image analysis was performed by ImageJ using the “particle analysis” tool on threshold adjusted optical fields. P < 0.05 of “12 h” *vs* all the other conditions.

To support the oxymetric measurements we carried out an imaging analysis of the mitochondrial membrane potential in PBLM cells by confocal microscopy. To this aim we used the mitotropic probe TMRE, which is a lipophilic cation accumulating in functional mitochondria driven by the ΔΨ_m_. Fig 7 displays representative pictures of PBLM exposed to 5, 12 and 20 hours to EMFs and the corresponding image analysis. It is shown that the TMRE-related fluorescence underwent a small transient increase at the 12 h time-point of EMF exposure to recover at the 20 h time-point, in agreement with the respirometric measurements. It has to be recalled that the relationship between respiratory rate and mitochondrial potential is non-ohomic [39–41], thus little changes are expected for the ΔΨ_m_. It is worthy noting that the tiny corpuscular inter-cellular fluorescent signal attributable to platelet mitochondria displayed a more clustered appearance in EMFs-exposed samples. This was indicative of a greater platelet aggregation, as compared with untreated samples, and confirmed the observations made in phase-contrast microscopy.

**Fig 8:**
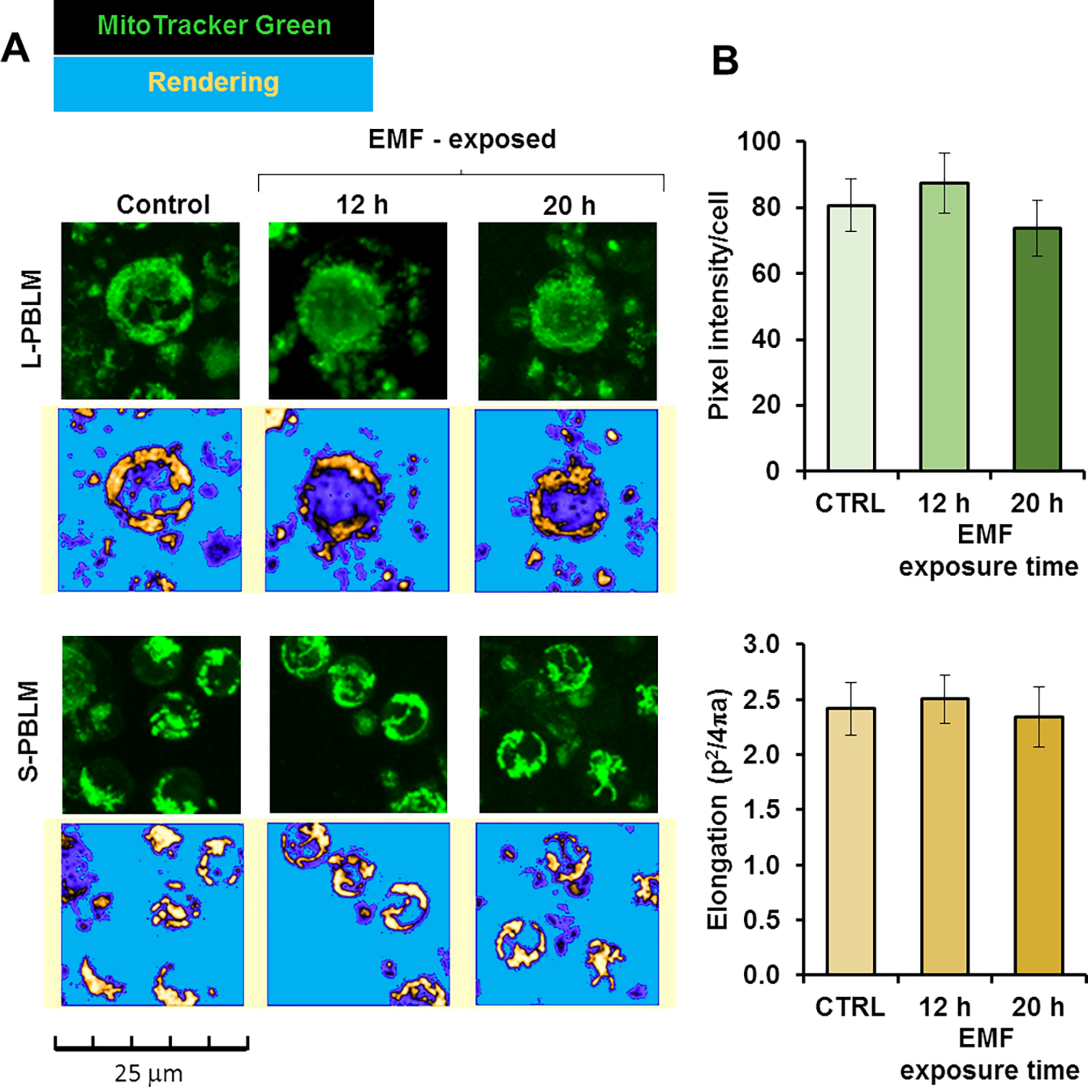
Confocal microscopy analysis of mitochondrial content in EMFs-exposed PBLM cells. PBLM cells exposed to EMFs for the indicated time were incubated with MitoTracker® Green and analyzed by confocal microscopy as detailed in Materials and Methods. (**A**) Representative images of cell subsets pinpointed on the basis of the size; L-PBLM and S-PBLM indicate large and small lympho-monocytes respectively. The picture below each microscopy image was attained by rendering of the corresponding fluorescence signal using ImageJ after removal of the back-ground. (**B**) Quantitative analysis of the MitoTracker®-related fluorescence/cell; the top histogram shows the fluorescence intensity/cell, the bottom histogram shows the elongation parameter of the fluorescent intracellular signal defined as the reciprocal of the circularity value. The bars are means ±SEM of 3 independent biological replicates; for each biological replicate the digitalized mean fluorescence/cell (ranging from 0 to 255) and the circularity of the signal was averaged from 50 to 100 single cell-analysis from at least three different optical fields for each condition. Image analysis was performed by ImageJ using the “particle analysis” tool on threshold adjusted optical fields.

**Fig 9:**
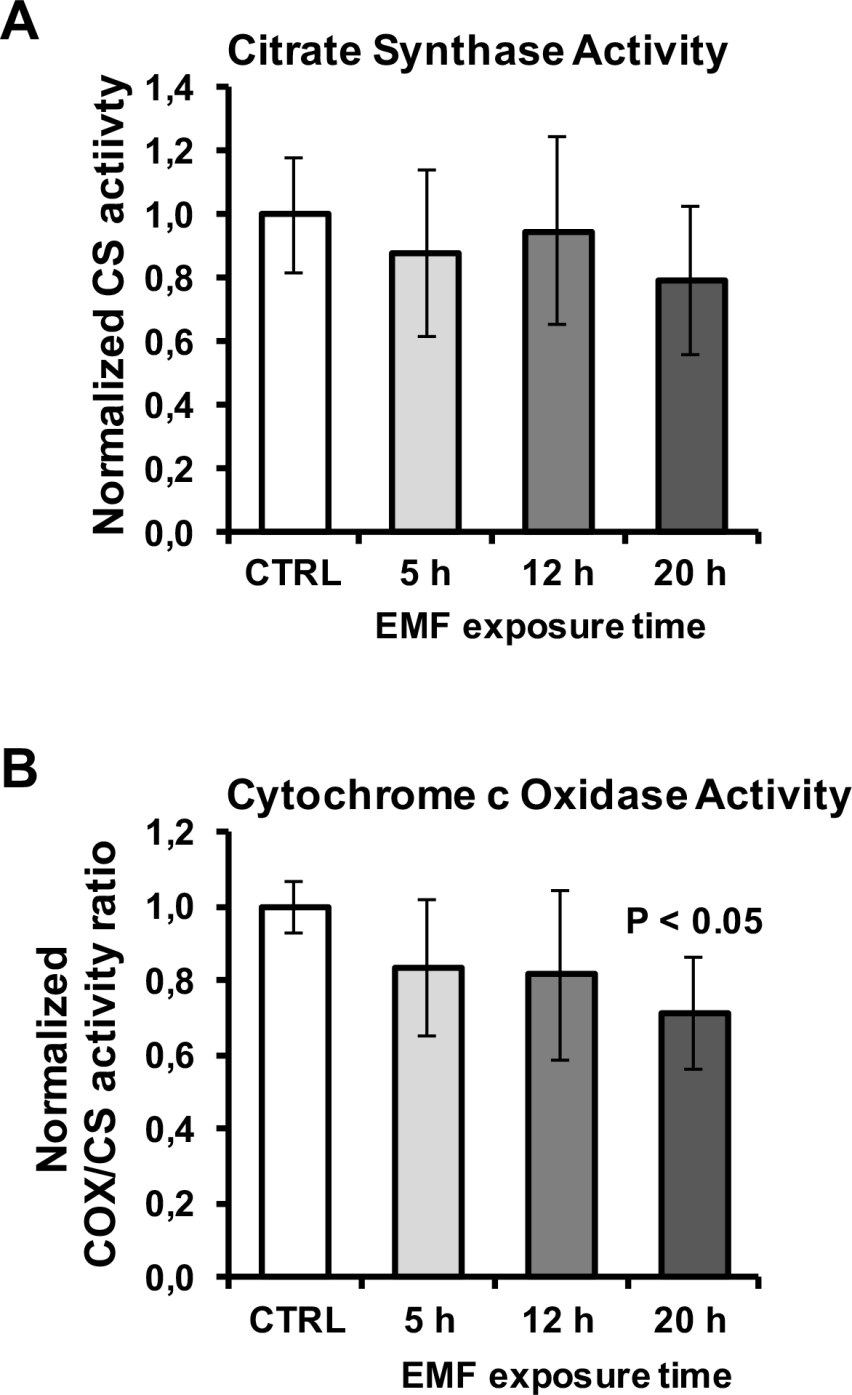
Measurement of mitochondrial markers in EMFs-exposed PBLM cells. The activity of citrate synthase (**A**) and of cytochrome c oxidase (**B**) was measured spectrophotometrically as detailed in Materials and Methods. The activity of citrate synthase was normalized to the cell number whereas that of cytochrome c oxidase to the citrate synthase. The values are means ±SEM of 3 biological replicates. P < 0.05 vs CTRL.

Next we assessed the overall mitochondrial content in PBLMs using the specific mitochondrial probe MitoTracker Green. Fig 8A shows selected representative images of large PBLM (about 12–15 μm diameter) and small PBLM (about 8–12 μm diameter) with condensed mitochondria distributed peri-nuclearly. Image analysis did not result in significant changes neither in the overall fluorescence/cell or in the shape of the intracellular fluorescence signal (Fig 8B). This indicated no appreciable variation either in the mitochondrial content and morphology after EMFs exposure. This conclusion was further supported by measuring the citrate synthase activity, an indirect marker of the mitochondrial mass, which did not display significant changes in EMFs-exposed PBLM (Fig 9A). Conversely the cytochrome c oxidase (COX) activity normalized to the citrate synthase activity resulted trendily to decrease though not reaching statistical significance even at the longest EMFs time exposure (Fig 9B).

**Fig 10:**
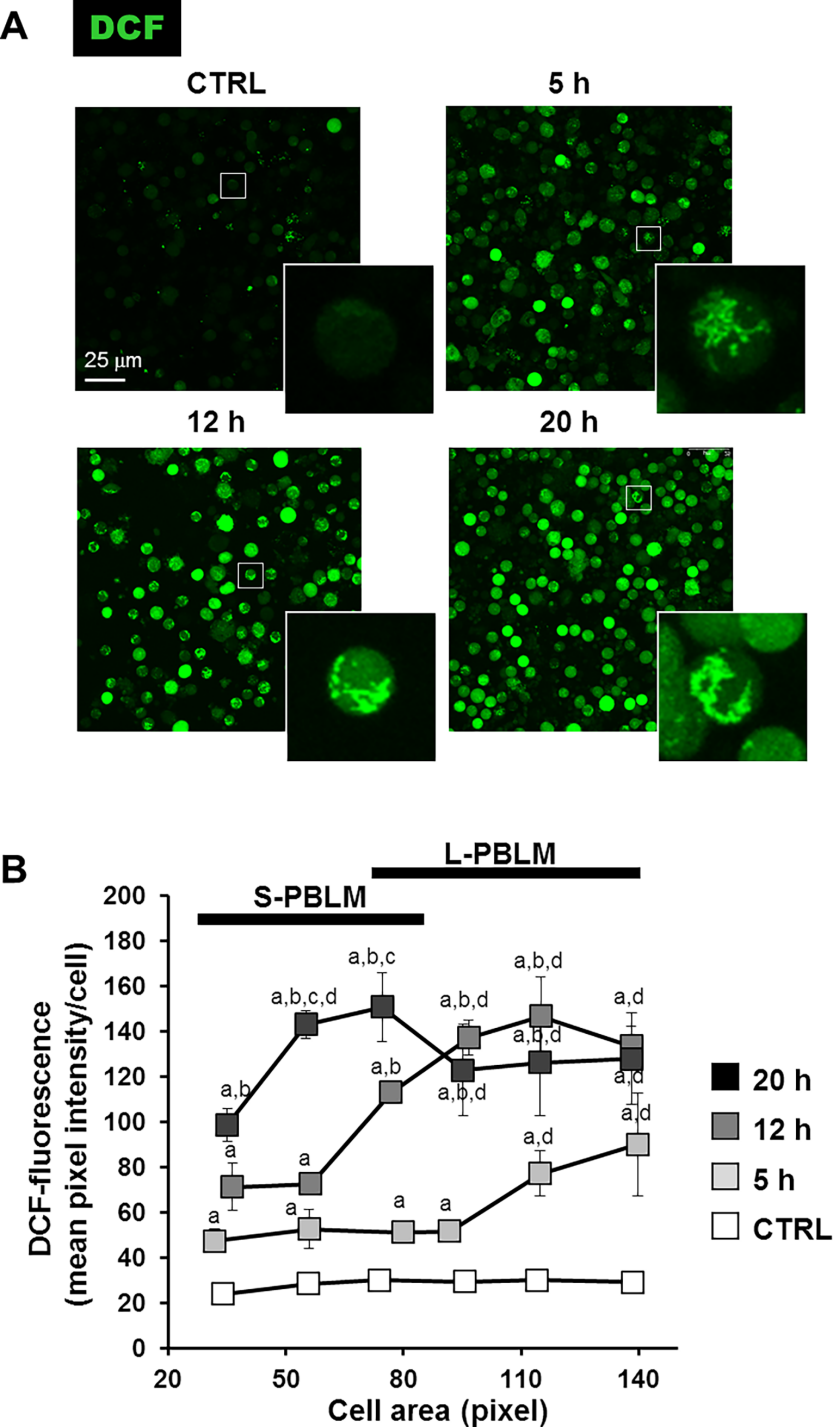
Confocal microscopy analysis of ROS production in EMFs-exposed PBLM cells. PBLM cells exposed to EMFs for the indicated time were incubated with 2,7-dichlorofluorescin diacetate and analyzed by confocal microscopy as detailed in Materials and Methods. (**A**) Representative images are shown with enlargement of selected cells. (**B**) DCF-stained PBLMs were clustered as a function of the calculated area and the fluorescence intensity/cell averaged (from 50 to 100 single cell-analysis under each condition). The values are means ±SEM of 3 biological replicates. The black bars in the graph denote the size range of the two major cell subsets indicated as small and large size PBLM (S-PBLM and L-PBLM respectively). Image analysis was performed in threshold adjusted optical fields using the “particle analysis” tool of ImageJ. Conditions for statistical significant difference (i.e. P < 0.05): “a”, vs CTRL of the same size cluster; “b”, 12 h vs 5 h of the same size cluster; “c”, 20 h vs 12 h of the same size cluster; “d”, L-PBLM vs S-PBLM subjected to the same EMF-exposure times.

Cytochrome *c* oxidase is the terminal complex of the respiratory chain delivering electrons to O_2_ which is reduced to 2 H_2_O molecules. Impairment of COX activity possibly causes production of reactive oxygen species because of the upstream accumulation of reducing equivalents in the electron transfer chain. The consequent risk of electron leakage to O_2_ produces the superoxide anion O_2_^•-^, which in turn is converted to H_2_O_2_ by the Mn-SOD. To assess this possibility PBLM cells were treated with the redox probe DCF after different time points of EFMs exposure. As shown in Fig 10A a significant increase of the DCF-related fluorescence signal was observed in EMFs-exposed PBLM cells, as compared with untreated cells, starting already after 5 h of treatment, which progressively enhanced at longer time-points of EMF challenging. Remarkably, the intracellular DCF fluorescence signal appeared to be partitioned in a subset of the cells clearly resembling the mitochondrial compartment (compare with Fig 8A). This points to mitochondria as the earlier ROS source then after diffusing in the rest of the cell. A closer image analysis unveiled that the progressive increase of DCF-related fluorescence as a function of the EMFs exposure time did not occur homogeneously in the PBLM. Indeed, it was shown that the larger was the size of the cell the earlier was the appearance of the DCF-related fluorescence (Fig 10B). Most notably, the fluorescence was evident only in PBLM cells and not in platelets which also contains mitochondria as shown in Figs 7 and 8. This last observation clearly indicates that EMFs exposure causes an unbalance of the cellular redox homeostasis occurring only in nucleated cells.

## Conclusions

This work investigates the effects of 1.8 GHz electromagnetic field at the electric field strength of 200 V/m on peripheral blood, focusing the analysis to leukocyte cells isolated from the blood after exposure to 1h, 5h, 12h and 20h times. The physical parameters used in the exposure protocol are within the EMFs values produced by mobile phones at a SAR comparable to the limit values allowed in European countries. Based on the discussed results, it is justified to conclude that the EMFs exposure of microwave radiation may alter the morphology of the lympho-monocyte blood component. Most notably, Raman spectroscopy analysis unveils changes in the biochemical composition of the lympho-monocyte nuclear compartment before the overt appearance of the cellular morphological changes. Such differences mainly consist of a decrease of the DNA and nucleic acid backbone-related signals with respect to those of the proteins, possibly caused by EMFs-induced alteration of the chemical environment surrounding the nucleus. Respirometric analysis of EMFs-exposed PBLM cells, largely constituted by lymphocyte, results in in a biphasic outcome with an earlier impairment of the mitochondrial OxPhos activity followed by an overwhelming recovering. This effect is paralleled by changes in the ΔΨ_m_. No appreciable variations either in the mitochondria mass and morphology is observed ruling out substantial changes in mitochondrial biogenesis or degradation. Nevertheless, a clear alteration of the intracellular redox balance is observed in the EMFs-treated nucleated PBLM preparations at time-point as earlier as 5 h. The impact of EMFs on the cellular ROS level has been object of extensive investigation leading to contrasting results. Multiple factors could cause these discrepancies, including but not limited to EMFs type/intensity/frequency, exposure time and assay time-point, as well as different biological samples examined [42]. The novel finding reported in the presented study would indicate the requirement of a functional nucleus in the cell to elicit the observed EMFs-mediated unbalance of the cellular redox tone since this was absent in the anucleate platelets.

The biophysical causes and biological consequences of such changes remain still unclear, because more statistically relevant samples are required to investigate effects on long time scale. Moreover, the obtained results refer to samples consisting of a mixture of monocyte and lymphocyte cells, although the fraction of the former component is larger with respect to the latter one. In order to complete the study, a proper isolation procedure of each one of the two components is needed to investigate if the EMFs exposure effects recur for each homogeneous cellular sample. Nonetheless, the original findings reported in this study suggest that EMFs exposure may affect the DNA and metabolic homeostasis in immune-competent cells potentially influencing both the innate and adaptive immune response and remark the need of a proper use of EMFs based-technologies to preserve the human health.
