# Correction: Exposure to 1.8 GHz electromagnetic fields affects morphology, DNA-related Raman spectra and mitochondrial functions in human lympho-monocytes

**DOI:** 10.1371/journal.pone.0198892

**Published:** 2018-06-07

**Authors:** M. Lasalvia, R. Scrima, G. Perna, C. Piccoli, N. Capitanio, P. F. Biagi, L. Schiavulli, T. Ligonzo, M. Centra, G. Casamassima, A. Ermini, V. Capozzi

The Funding section is incorrect. The correct funding information is: Published with a contribution from 5 x 1000 IRPEF funds in favor of the University of Foggia, in memory of Gianluca Montel.
